# Methodological issues in the development of a prediction model for pre-eclampsia complications

**DOI:** 10.1186/1745-6215-14-S1-P30

**Published:** 2013-11-29

**Authors:** Ewelina Rogozinska, Fiona Fong, Shakila Thangaratinam, John Allotey, Julie Dodds, Sally Kerry, Nadine Marlin, Khalid Khan

**Affiliations:** 1 Women's Health Research Unit, London, UK; 2 Centre for Primary Care and Public Health, London, UK

## 

PREP (Prediction of risks in early onset pre-eclampsia) is a multicentre prospective cohort study aiming to develop and validate a prediction model in women admitted with early-onset pre-eclampsia for risk of adverse maternal outcome at 48 hours and until discharge. The primary outcome was a composite of adverse maternal outcomes and the secondary outcome a composite of adverse fetal outcomes.

The planned model includes multiple predictor variables in combination to predict the risk of adverse outcomes in individual patients. We highlight the methodological challenges encountered in model development.

The predictor variables were pre-selected based on symptoms, signs and results of investigations routinely taken during management of pre-eclampsia. Challenges included accounting for correlation between variables, choosing the most abnormal variable within a time period or from a pre-determined time point, inconsistency in ascertainment of the data points ie blood results available later than the signs and symptoms, modelling a combination of binary, ordinal and continuous variables and the approach in prioritisation of these variables.

Clinician management such as anti-hypertensives and magnesium sulphate will affect the relationship between the predictor and outcome. We will include clinical management as a covariate and assess consistency in model performance across different treatment groups to account for this.

Selecting the optimum type of prediction model posed a further challenge. Logistic regression focuses on the relationship between predictor variables and adverse outcomes whereas survival analysis takes into account the time to develop the outcome. The former may be statistically more suitable but the latter clinically more useful.

